# Neuroprotective Effects of Aged Garlic Extract on Cognitive Dysfunction and Neuroinflammation Induced by β-Amyloid in Rats

**DOI:** 10.3390/nu9010024

**Published:** 2017-01-03

**Authors:** Nutchareeporn Nillert, Wanassanun Pannangrong, Jariya Umka Welbat, Wunnee Chaijaroonkhanarak, Kittisak Sripanidkulchai, Bungorn Sripanidkulchai

**Affiliations:** 1 Department of Anatomy, Faculty of Medicine, Khon Kaen University, Khon Kaen 40002, Thailand; nutchareeporn16@gmail.com (N.N.); wankun@kku.ac.th (W.P.); jariya@kku.ac.th (J.U.W.); cwunnee@kku.ac.th (W.C.); skitti@kku.ac.th (K.S.); 2 Center for Research and Development of Herbal Health Products, Khon Kaen University, Khon Kaen 40002, Thailand; 3 Neuroscience Research and Development Group, Khon Kaen University, Khon Kaen 40002, Thailand

**Keywords:** aged garlic extract, Alzheimer’s disease, β-amyloid, neuroinflammation, neuroprotection, object recognition

## Abstract

Neuroinflammation is pathological evidence of Alzheimer’s disease (AD) that likely starts as a host defense response to the damaging effects of the β-amyloid (Aβ) deposits in the brain. The activation of microglia may promote the neurodegenerative process through the release of proinflammatory cytokines, such as interleukin-1β (IL-1β) and tumor necrosis factor-α (TNFα), which may lead to neuronal damage and eventual death. Aged garlic extract (AGE) has been reported to have multiple biological activities, including anti-inflammatory effects. Therefore, the objective of this study was to investigate the effect of AGE on Aβ (1-42)-induced cognitive dysfunction and neuroinflammation. Adult male Wistar rats were given AGE (125, 250, and 500 mg/kg BW, body weight), orally administered, daily for 56 days. They were then injected with 1 μL of aggregated Aβ (1-42) into the lateral ventricles; bilaterally. Seven days later, their recognition memory was evaluated using a novel object recognition (NOR) test. Then the rats were sacrificed to investigate the alteration of microglia cells, IL-1β and TNFα in the cerebral cortex and hippocampus. The results indicated that AGE at doses of 250 and 500 mg/kg BW significantly improved short-term recognition memory in cognitively impaired rats. In addition, AGE significantly minimized the inflammatory response by reducing the activation of microglia and IL-1β to the levels found in the control, which is similar to the results found in Celebrex-treated rats. In conclusion, AGE may be useful for improving the short-term recognition memory and relieve the neuroinflammation in Aβ-induced rats.

## 1. Introduction

Alzheimer’s disease (AD) is the most common form of dementia. It is clinically diagnosed using the following criteria: progressive loss of cognition and memory, alterations of personality, and decreasing visual skills [1]. β-amyloid (Aβ) formation in vulnerable brain regions, such as the hippocampus and cerebral cortex, is a major neuropathological feature of AD [2,3]. It is believed that the deposition of Aβ triggers a series of inflammatory processes, which likely start as a host defense response to the damaging of tissue, and later contributes to neuronal degeneration [2,4]. The activation of microglia may promote the neurodegenerative process through the release of proinflammatory cytokines, such as interleukin-1β (IL-1β) and tumor necrosis factor-α (TNFα) [5,6,7], and other toxic products [8], which may lead to neuronal cell damage and eventual death. Furthermore, these inflammatory mediators present in AD lesions are thought to stimulate the key events of the pathological cascade that result in an increase of Aβ production, which includes the recruitmentand activation of microglia cells [9]. A current treatment for AD is aimed at reducing amyloid formation by restoring cholinergic deficits with the use of cholinesterase inhibitors (e.g., Donepezil and Rivastigmine) and by regulating neuroinflammation with the use of COX-2 inhibitors (e.g., Celebrex) and non-steroidal anti-inflammatory drugs (NSAIDs, e.g., ibuprofen and indomethacin). However, most of these drugs can cause various side effects such as liver and renal toxicity, gastro-intestinal bleeding, and nausea. In this respect, natural herbal sources with the least amount of adverse effects may provide greater therapeutic benefit in terms of reduction or prevention of neuroinflammation. They could, thus, be beneficial in the treatment of neurodegenerative diseases, including AD. Garlic (Allium sativum) has been recognized for its medicinal value. For this study, fresh garlic was aged over a prolonged period of time in order to create aged garlic extract (AGE). AGE produces a rich content of stable organosulfur compounds such as S-allyl cysteine (SAC) [10], and many other thiosulfinates that are known to exert multiple benefits, e.g., anti-oxidant, anti-inflammatory, and anti-apoptotic effects [11]. Over 350 scientific studies have shown that AGE is safe and effective in providing health benefits in humans [12]. Its neuroprotective effect has also been evaluated in an animal model [13]. AGE has the potential to protect the brain against neurodegenerative conditions [14,15,16,17,18] by preventing brain injury following ischemia [18], protecting neuronal cells against apoptosis [14,17,19], and preventing β-amyloid-induced oxidative death [15,16]. Moreover, treatment with AGE or S-allyl cysteine has been shown to prevent the degeneration of the brain’s frontal lobe, improve learning and memory retention, and extend lifespan [20,21]. However its anti-inflammatory effect against β-amyloid-induced toxicity in rats has not yet been investigated. Therefore, the present study used various doses of AGE to demonstrate its beneficial effects on memory performance and against the neuronal toxicity in the hippocampus after Aβ-induced microglia activation in animal models.

## 2. Materials and Methods

### 2.1. Animals

Healthy adult male Wistar rats, 180–220 g in weight, were obtained from the National Animal Center, Mahidol University, Thailand. All experiments were conducted under the National Guidelines of Animal Care and were approved by the Ethics Committee of Khon Kaen University (Approval No. 0514.1.12.2/64). The rats were housed at 23 ± 2 °C under a 12 h light/12 h dark cycle (lights on from 06:00 to 18:00). They were maintained in groups of four in cages with free access to food and water.

### 2.2. Materials and Plant Extract Preparation

The aged garlic extract (AGE) was provided by the Center for Research and Development of Herbal Health Products (CRD-HHP), Khon Kaen University, Thailand. AGE was prepared from fresh garlic in 30% ethanol and fermented at room temperature for approximately 15 months under light protection. AGE used for this study contained S-allylcysteine (SAC) at 30.96 mg/g and allicin at 32 µg/g (Petty Patent No. 3506, Thailand). All chemicals in this study were analytical grade.

### 2.3. Aβ (1-42) Injection and Drug Treatments

**Figure 1:**
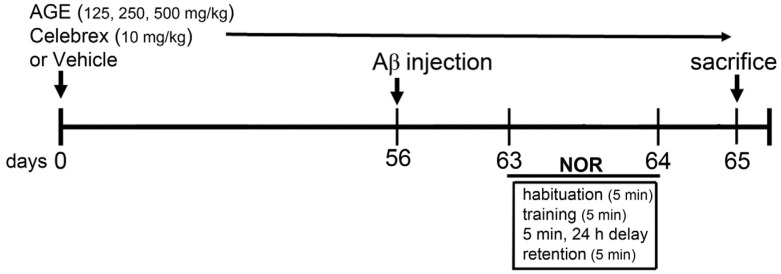
A schematic diagram of drug treatment and behavioral tests. Rats were injected with Aβ (1-42) into both sides of lateral ventricle after 56 days of drug treatments (NOR: novel object recognition, Aβ: β-amyloid (1-42)).

Aβ (1-42) peptide (Alexis Biochemicals, San Diego, CA, USA) was dissolved in glacial acetic acid at a concentration of 1 µg/µL, and the solution was incubated at 37 °C for 24 h to induce peptide aggregation. The aliquots were stored at −20 °C until the moment of use. Forty-eight rats were randomly divided into six groups (*n* = 8). Group 1 was the control group, consisting of healthy rats that did not receive any treatment. The rats in Group 2 (V + Aβ) were fed distilled water. Those in Group 3 (Celeb + Aβ) were fed Celebrex at 10 mg/kg BW. Finally, the rats in Groups 4, 5, and 6 (AGE125 + Aβ, AGE 250 + Aβ and AGE500 + Aβ) were fed with AGE at 125, 250, and 500 mg/kg BW, respectively. The treatments were administered daily for sixty-five days. At day fifty-six, all rats in Groups 2–6 were injected with 1 μL of aggregated Aβ (1-42) peptide into each lateral ventricle, bilaterally at a rate of 0.2 μL/min [22] using the following coordinates: AP −0.8 mm from bregma; ML ± 1.5 mm from bregma; SI −3.8 mm from dura [23]. Seven days after Aβ (1-42) injection, all rats were tested for novel object recognition (NOR) (Figure 1). They were then sacrificed and the density of microglia and concentration of IL-1β and TNFα in the cerebral cortex and hippocampus were determined.

### 2.4. Novel Object Recognition (NOR)

The NOR procedure consisted of three sessions: habituation, training, and retention. In the first session, each rat individually explored the box, which was empty of any objects, for 5 min. During the training session, two novel objects were introduced, each placed at a different location in the box. Each rat was then allowed to explore in the box again for 5 min. A rat was considered to be exploring an object when its head was facing the object or it was touching or sniffing the object. The time spent exploring each object was recorded. After training, the rats were immediately returned to their home cages. In the last session, one of the objects was replaced with a novel object. Twenty-four hours after the five-minute training session, each rat was again placed in the box to explore for 5 min, and the time spent exploring each object was recorded. The box and objects were cleaned with 20% ethanol before the first trial and between trials to minimize odor cues. The ability to recognize the novel object was evaluated using a discrimination index (DI) calculated for each animal using the formula: (N − F/N + F) [24], which corresponds to the difference between the time exploring the novel (N) and the familiar object (F), corrected for total time exploring both objects. The result can vary between +1 and −1, where a positive score indicates more time spent with the novel object, a negative score indicates more time spent with the familiar object, and a zero score indicates a null preference.

### 2.5. Tissue Processing

After the behavioral tests, the rats were anesthetized and perfused with 0.9% normal saline. Brains were immediately put on ice and cut at the midline dividing the brain into two hemispheres. The cerebral cortex and hippocampus from the right hemisphere were separated and immediately stored at −80 °C for Western blotting analysis. The left hemisphere was cryopreserved in sucrose solution (30%) and fixed in ice-cold 4% paraformaldehyde solution for immunohistochemical investigation of anti-integrin αM (CD11b) using a free-floating technique. Coronal serial sections (35 µm thick) were cut with a sliding microtome and put in cold 0.01 M phosphate buffer solution, pH 7.4 (PBS).

### 2.6. Immunohistochemistry

Coronal brain sections from each animal were incubated in blocking serum (1% bovine serum albumin (BSA) in PBS) for 1 h. They were incubated overnight with mouse monoclonal anti-integrin αM (CD11b) antibody (1:100; Merck Millipore, Darmstadt, Hesse, Germany) in blocking serum. After rinsing, the sections were incubated with HRP-goat anti-mouse IgG secondary antibody (1:500; Invitrogen, Carlsbad, CA, USA) at room temperature for 2 h. After washing in PBS, the sections were treated with 0.001% diaminobenzidine tetrahydrochloride dihydrate (Sigma-Aldrich, St. Louis, MO, USA) in PBS containing 0.003% H_2_O_2_ (Merck, Darmstadt, Germany). Then the sections were mounted on gelatin-coated glass slides, allowed to dry overnight, dehydrated and cover-slipped under DPX (Sigma, St. Louis, MO, USA). Slides were viewed under a light microscope (Nikon Microscope ECLIPSE E200 MVR) with a 40× objective, and microglia positive cells were blindly counted in the hippocampus and cerebral cortex. The immunostaining was analyzed as the percentage of labeled area captured (positive pixels)/the full area captured (total pixels) using the image processing and analysis using Java software (Windows version, National Institutes of Health, Bethesda, MD, USA) [25,26,27].

### 2.7. Western Blotting

The brain tissue was prepared for Western blotting, as has previously been described [28]. Protein samples were resolved on 12% sodium dodecyl sulfate polyacrylamide gel electrophoresis under reducing conditions (Bio-Rad Laboratories GmbH, Munich, Germany). They were electrophoretically transferred onto nitrocellulose membranes and blocked with 5% non-fat dried milk (Sigma-Aldrich) in 25 mM tris-buffered saline containing 0.1% tween 20 (TBS-T) at room temperature for 1 h. The membranes were then probed with primary antibodies, including a rabbit polyclonal anti-IL1-β antibody (1:500; Abcam, Milton, Cambridge, UK), a rabbit polyclonal anti-TNFα antibody (1:100; Abcam, Milton, Cambridge, UK) and a mouse monoclonal anti-glyceraldehyde 3 phosphate dehydrogenase (GAPDH) antibody as a reference protein (1:20,000; Abcam, Milton, Cambridge, UK) in TBS-T at 4 °C for 24 h. After extensive washing with TBS-T, the membranes were incubated with peroxidase conjugated secondary antibody (1:1000, Merck Millipore, Billerica, MA, USA) at room temperature for 2 h. Signals were detected by enhanced chemiluminescence (Thermo Scientific, Waltham, MA, USA) and exposed onto film. The optical density of the bands was quantified using the image processing and analysis with Java software (Windows version, National Institutes of Health, Bethesda, MD, USA).

### 2.8. Statistical Analysis

All statistical parameters were calculated using GraphPad Prism (V 5.0, GraphPad Software, La Jolla, CA, USA). All data are expressed as means ± standard error of mean (S.E.M.). Statistical significance was determined using one-way analysis of variance (ANOVA) followed by a Newman-Keuls post-hoc test for multiple comparisons. Differences were considered significant at *p* < 0.05.

## 3. Results

### 3.1. Effect of AGE On Recognition Memory

**Figure 2:**
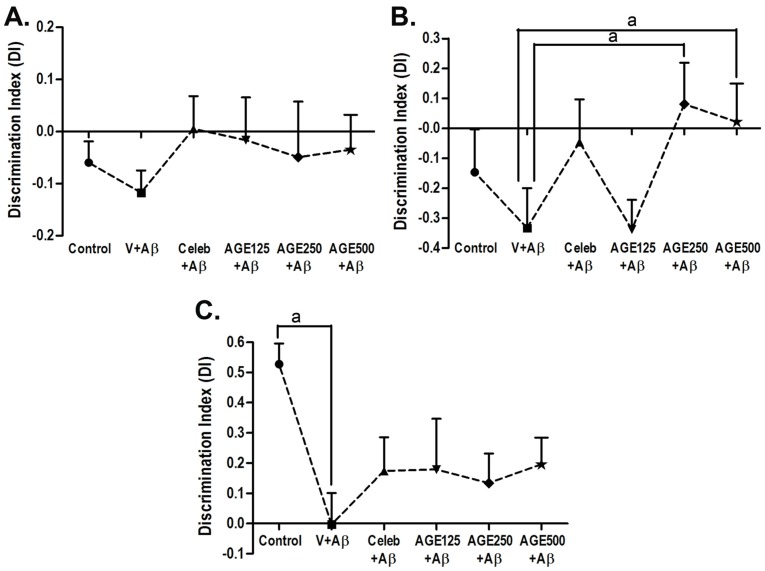
The discrimination index (DI) in the training phase (**A**); after a five-minute delay (**B**) and 24 h delay (**C**) in the novel object recognition test. Data are presented as mean ± S.E.M. (standard error of mean), a = significant differences from vehicle + Aβ group at *p* < 0.05.

Recognition memory was evaluated by NOR at day 63 and day 64 (Figure 2). During training session, the results shows no significant difference in the time spent exploring the two identical objects, because both objects were novel (Figure 2A). In the first retention period (delay 5 min), only the animals that received Aβ (1-42) and AGE125 still showed deficits in short-term memory retention. In contrast, the animals given AGE at doses of 250 and 500 mg/kg BW explored the novel object significantly more than the familiar object (a, *p* < 0.05; Figure 2B), indicating recognition memory retrieval. However, in the long-term retention test (delay 24 h), all doses of AGE tended to increase the discrimination index, but did not reverse the toxic effect of Aβ (1-42), as observed by the fact that there were no significant differences among the groups in time spent exploring the novel object. The group that received Aβ (1-42) showed significant memory deficit when compared to the control (a, *p* < 0.05; Figure 2C).

### 3.2. Effect of AGE on Microglia Activation in the Cerebral Cortex and Hippocampus

**Figure 3:**
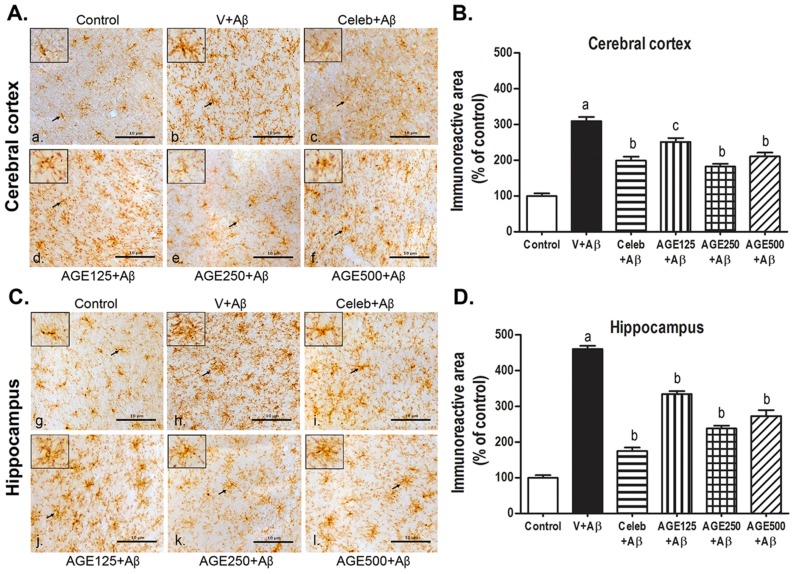
Effect of AGE on CD11b immunoreactivity in the cerebral cortex and hippocampus of the rat brain: (**A**,**C**) the photomicrographs of brain sections showing the distribution of microglia activating cells by immunohistochemistry staining in the cerebral cortex and hippocampus, respectively; Control (**a**,**g**); V + Aβ (**b**,**h**); Celeb + Aβ (**c**,**i**); AGE125 + Aβ (**d**,**j**); AGE250 + Aβ (**e**,**k**) and AGE500 + Aβ (**f**,**l**); arrows point to examples of microglia cells in the rectangular frame; (**B**,**D**) The bar graphs represent the CD11b immunoreactive area in the cerebral cortex and hippocampal regions of the rat brain. Data are presented as mean ± S.E.M., a = significant differences from control group at *p* < 0.001 and b, c = significant differences from vehicle + Aβ group at *p* < 0.001 and *p* < 0.01, respectively.

CD11b is a member of the integrin family that expresses on the surface of microglia. Increased expression of CD11b represents microglia activation during neurodegenerative inflammation. In this study, the determination of CD11b immunoreactivity was performed as a measure of microglia activation. Microglia in two regions of the control brain sections were generally found to have a ramified morphology (Figure 3a,g). In Aβ (1-42) treated rats, the microglia showed various clusters of cells with shortened and thickened processes similar to amoeboid morphology, indicating activated microglia (Figure 3b,h). Some microglia remained ramified with small cell bodies, and some became hypertrophic with strongly stained cell bodies and thicker processes were seen in the AGE-(Figure 3d–f,j–l) and Celebrex-treated rats (Figure 3c,i). Some aggregation of microglia could also be observed in the AGE- and Celebrex-treated rats. The CD11b immunoreactivity in the cerebral cortex and hippocampus were also analyzed as percentage of labeled area. They were significantly higher in the Aβ (1-42)-treated groups when compared to the control group (a, *p* < 0.001; Figure 3B,D). However, the activation of microglia was significantly inhibited after Aβ (1-42) injection in animals treated with Celebrex and all doses of AGE (b, *p* < 0.001 and c, *p* < 0.01; Figure 3B,D) in both regions of the brain, compared with those animals treated with saline.

### 3.3. Effect of AGE on IL-1β and TNFα Expression in the Cerebral Cortex and Hippocampus

**Figure 4:**
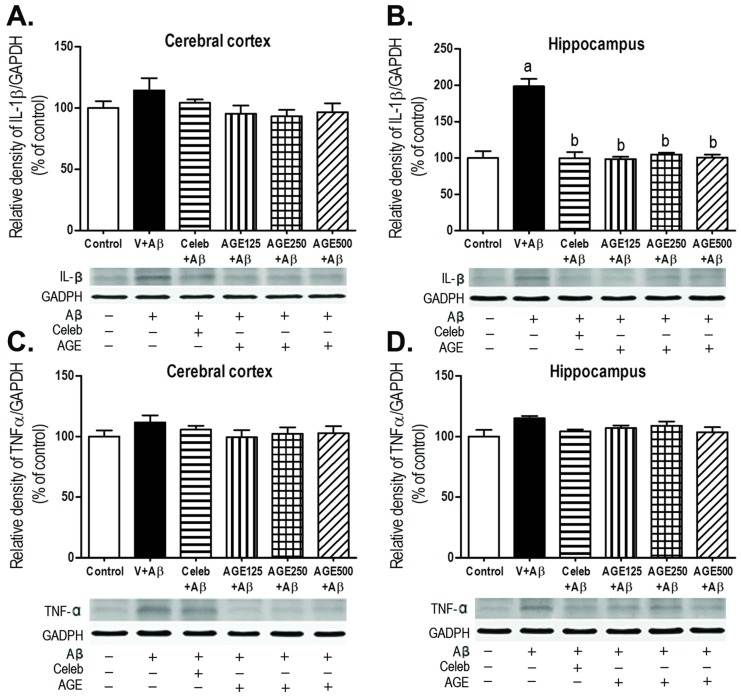
Effects of AGE on IL-1β (**A**,**B**), and TNFα (**C**,**D**) densities in the rat brain. Data are presented as mean ± S.E.M. a = significant differences from control group at *p* < 0.001 and b = significant differences from vehicle + Aβ group at *p* < 0.001.

Activated microglia cells are significant sources of proinflammatory cytokines and neuroinflammation. This study investigated the density of two proinflammatory cytokines, IL-1β and TNFα, induced by Aβ (1-42) peptides using Western blotting analysis. Treatment with Aβ (1-42) significantly increased the density of IL-1β in the hippocampus, but not in the cerebral cortex when compared to control group (a, *p* < 0.001; Figure 4A,B), whereas the density of TNFα in both regions of the rat brain (Figure 4C,D) did not change. The treatment of AGE at all doses markedly decreased the up-regulation of IL-1β in the hippocampal region of the rat brain in a similar fashion as did Celebrex (b, *p* < 0.001; Figure 4B).

## 4. Discussion

Our findings revealed that pre-exposure to all doses of AGE prevented recognition memory impairment caused by Aβ (1-42)-treatment. AGE at doses of 250 and 500 mg/kg BW can ameliorate cognitive impairments in short-term memory (5 min delay) (Figure 2B), but all doses of AGE were not able to significantly improve long-term memory in the novel object recognition test after a 24 h delay (Figure 2C). As previously reported, the rats generally spend more time exploring new objects than they do to familiar objects when the interval between the sample trial and the choice trial is 1 h or less. However, if the interval is longer, the rats cannot differentiate the familiar and new objects [29,30,31]. AGE pretreatment can bring back learning and recognition memory, as detected in the 5 min delay where the Aβ-induced rats spent more time to explore the new objects than the familiar objects. It is suggested that AGE is able to alleviate cognitive impairment involving short-term memory. However, the fact that the impairment in long-term memory is not encountered by AGE pretreatment in this study may reflect to the incomplete recovery of hippocampus injury from Aβ-induction. Since the delay-dependent decline in recognition memory is related to the perirhinal cortex to maintain information about the object during longer retention intervals [32,33], we observed that the injury is also confined to this region of the brain (data not shown). In addition, the intracerebroventricular (ICV) injection of Aβ into the rat ventricle activated the microglia cells [34] and there is evidence to suggest that microglia activation can produce pro-inflammatory cytokines and reactive oxygen species (ROS) [35] causing peroxidation of membrane lipids and neuronal death [36,37] in normal aging and neurodegenerative diseases.

Moreover, stimulation of microglia can increase the CD11b expression in several neurodegenerative diseases [38]. The present data revealed that Aβ (1-42) significantly increased the density of CD11b-positive microglia immunoreactivity in both the cerebral cortex and hippocampus when compared to the control. AGE at all doses (125, 250, and 500 mg/kg BW) were effective against the inflammatory response induced by Aβ (1-42) in that they dramatically decreased the density of CD11b-positive microglia immunoreactivity in both the cerebral cortex and hippocampus. These findings are in concordance with previous report that chronic administration of AGE and SAC can reduce the number of activated microglia surrounding Aβ plaques in an Alzheimer’s Swedish double mutant mouse (Tg2576) [39]. Furthermore, chronic activation of microglia can lead to an inflammatory response through the release of various proinflammatory cytokines, such as IL-1β and TNFα [40]. These cytokines are fundamental indicators of the inflammation processes [41] and overexpression of IL-1β surrounding Aβ plaques was observed in the AD brains [42,43,44]. IL-1β has also reported to increase the production and accumulation of Aβ [45,46,47]. This study showed that ICV injection of Aβ (1-42) significantly increased the density of IL-1β in the hippocampus but not in the cerebral cortex. This is probably explained by the fact that the hippocampus is near to the location of the Aβ (1-42) injection than cerebral cortex and, thus, Aβ caused more direct neurotoxicity to the hippocampus [48]. We did not find the changes in the level of another proinflamatory cytokine, TNFα, in both the cerebral cortex and hippocampus of the brains in all studied groups. Similarly, the previous studies had shown that microglia activation did not induce TNFα protein level in mice with lesion-induced axonal degeneration [49] and the serum levels of TNFα did not change in olfactory bulbectomized-induced rats [50]. In terms of the mechanism of action, Aβ interacts with several receptors on the cell surface of microglia, especially CD36. The combination of Aβ with CD36 receptors, CD36 acting as a co-receptor of TLR4 and TLR6 expressed in the form of CD36-TLR4-TLR6 complex [51], stimulates the NOD-, LRR-, and pyrin domain-containing 3 (NLRP3) inflammasome to change to NLRP3 inflammasome activation signaling, inducing the release of IL-1β to come outside the microglia [52]. Therefore, Aβ injection induced more severe inflammation in the hippocampus than in cerebral cortex, resulting in the significant release of IL-1β in this region of the brain. While TNFα were significantly produced by activation of monocytes and the severity of inflammation from Aβ-treatment is not enough to induce the infiltration of monocytes into brain tissue [53]. The finding on neuroprotective and anti-inflammatory effects of AGE in our studies is in concordance with several previous reports, such as AGE and SAC (a major compound from AGE) decreased the brain levels of IL-1β and TNFα in Tg2576 mice [39], SAC protected the cell death of Aβ (1-40)-induced PC12 cells [54], the aqueous garlic extracts reduced the release of prionflammatory cytokine (IL-1β and TNFα) in lipopolysaccharide-activated RAW 264.7 cells [55,56], and allicin decreased the expression of prionflammatory cytokine (IL-1β and TNFα) in the traumatic brain injury in rats [57]. In terms of phytochemical analysis of AGE, SAC was detected as the major ingredient (30.96 mg/g) with low level of allicin (32 µg/g) [58]. Allicin is mainly found in fresh crushed garlic, is chemically unstable, and changed to SAC during the aging processes [56,59]. Therefore, SAC may be one of the chemical contributors of AGE to confer anti-inflmmatory effects against neurotoxicity of Aβ in our study. However, other compounds in AGE may also play roles and the mechanism of AGE in modulating neuroinflammaion is required to be investigated further.

In addition, several studies reported that the neuroprotective effects of natural products in AD models such as pretreatment of Cereboost™, an American ginseng extract, improved learning and memory-deficit by enhancing the brain acetylcholine level via ChAT gene expression in Aβ (1-42)-induced mice [60], treatment of curcumin decreased the prionflammatory cytokine, IL-1β, oxidative damage, insoluble Aβ, soluble Aβ, and Aβ plaque burden in Tg2576 mice [61], and treatment of green tea reduced the cerebral Aβ levels in Tg2576 mouse by promoting the non-amyloidogenic α-secretase proteolytic pathway [62]. The previous data demonstrate that several natural products are able to protect the nervous system and AGE is one of the potential candidates to delay the progression of AD.

## 5. Conclusions

In conclusion, ICV administration of Aβ (1-42) induced amnesic effects with regard to both short-term and long-term memories in rats. In addition, Aβ (1-42) caused an inflammatory response by activating the microglia and increasing the density of proinflammatory cytokine, IL-1β, in the rat brain. Pretreatment of AGE alleviated the recognition memory impairment involving short-term memory in Aβ (1-42)-induced rats by decrease the density of CD11b-positive microglia immunoreactivity and the density of IL-1β in the injured brain. Therefore, it is suggested that AGE could be a good supplementary food for the improvement of cognitive function in the elderly and AD patients.

## Abbreviations

The following abbreviations are used in this manuscript:

Aβ	β-amyloid
AD	Alzheimer’s disease
AGE	Aged garlic extract
ANOVA	analysis of variance
AP	anterior-posterior
BSA	bovine serum albumin
BW	body weight
Celeb	Celebrex
CD11b	anti-integrin αM
COX-2	cyclooygenase-2
CRD-HHP	Center for Research and Development of Herbal Health Products
DI	discrimination index
DPX	Depex mounting medium
F	familiar object
G	Gram
GAPDH	anti-glyceraldehyde 3 phosphate dehydrogenase
H	Hour
H_2_O_2_	hydrogen peroxide
ICV	Intracerebroventricular
IL-1β	interleukin-1β
Kg	Kilogram
M	Molar
Mg	Milligram
Min	Minute
MO	Missouri
μL	Microliter
µm	Micrometer
ML	medial-lateral
N	novel object
NLRP3	NOD-, LRR- and pyrin domain-containing 3
NOR	novel object recognition
NSAIDs	non-steroidal anti-inflammatory drugs
PBS	phosphate buffer solution
ROS	reactive oxygen species
SAC	S-allyl cysteine
S.E.M.	standard error of mean
SI	superior-inferior
TBS-T	tris-buffered saline containing 0.1% tween 20
TLR	Toll-like receptor
TNFα	tumor necrosis factor-α
USA	United States of America
V	Vehicle
